# Seasonal effects on ruminal fermentation and greenhouse gas emission patterns in non-lactating crossbred Saanen goats under tropical conditions: Evidence from respiratory chamber measurements

**DOI:** 10.14202/vetworld.2026.3337-3350

**Published:** 2026-08-04

**Authors:** Nhung Thi Cam Pham, Trung Thanh Truong, Thiet Nguyen, Narongsak Chaiyabutr, Sumpun Thammacharoen

**Affiliations:** 1 Department of Physiology, Faculty of Veterinary Science, Chulalongkorn University, Henri Dunang Street, Bangkok 10330, Thailand.; 2 Faculty of Animal Science, College of Agriculture, Can Tho University, Can Tho City, Vietnam.; 3 The Academy of Science, The Royal Society of Thailand, Dusit, Bangkok 10300, Thailand.; 4 Queen Saovabha Memorial Institute, The Thai Red Cross Society, Bangkok 10330, Thailand.

**Keywords:** crossbred Saanen goats, enteric methane emissions, greenhouse gas emissions, high ambient temperature, respiratory chamber, ruminal fermentation, seasonal heat stress, tropical conditions

## Abstract

**Background and Aim:**

High ambient temperature (HTa) associated with climate change poses challenges to livestock production and exacerbates greenhouse gas (GHG) emissions in a self-perpetuating cycle. While HTa influences ruminal fermentation and enteric methane (CH₄) emissions in dairy cows, data on long-term seasonal HTa effects in heat-adapted tropical dairy goats under natural conditions remain limited. This study aimed to evaluate seasonal HTa effects on ruminal fermentation patterns, physiological responses, and daily CO₂ and CH₄ emissions in non-lactating crossbred Saanen goats using respiratory chamber measurements. The research gap addressed includes the scarcity of studies decoupling long-term acclimatization from short-term diurnal HTa using paired ambient and cooled chamber conditions in small ruminants under true tropical environments.

**Materials and Methods:**

Twelve nulliparous crossbred Saanen × Bach Thao goats were used in a completely randomized design with two seasonal periods (winter: December–February; summer: March–May) and six replicates. Goats were housed individually and fed a total mixed ration (80% corn silage, 20% concentrate). Ambient conditions, dry matter intake (DMI), water intake (WI), rectal temperature (Tr), respiratory rate (RR), plasma cortisol, and ruminal volatile fatty acids (VFAs) were measured. Daily CO₂ and CH₄ emissions were quantified over 24 h in respiratory chambers under ambient and 25°C air-conditioned conditions at mid-experiment points. Data were analyzed using linear mixed-effects models with season, chamber, and their interaction as fixed effects.

**Results:**

Summer daytime temperature–humidity index exceeded 80, with a seasonal ΔTa of 3.0°C. Long-term HTa increased total ruminal VFA concentration (53.2 ± 0.7 vs. 57.8 ± 1.0 mM, p < 0.05) and acetate:propionate ratio (2.3 ± 0.2 vs. 3.3 ± 0.4, p < 0.05) while decreasing NH₃-N (30.1 ± 1.0 vs. 26.1 ± 0.1 mg/dL, p < 0.05), without affecting DMI or WI. Daily CO₂ (378 ± 40 vs. 407 ± 32 g/day) and CH₄ (7.2 ± 1.7 vs. 9.2 ± 1.0 g/day) emissions did not differ between seasons (p > 0.05). However, attenuating daytime HTa to 25°C reduced nighttime CO₂ emissions (188 ± 12 vs. 160 ± 5 g/day, p < 0.05), linked to altered drinking behavior.

**Conclusion:**

Seasonal HTa under tropical conditions altered ruminal fermentation without changing daily enteric CO₂ or CH₄ emissions in crossbred dairy goats, highlighting their superior adaptability. Short-term HTa attenuation revealed subtle effects on nighttime CO₂ via drinking behavior. These findings provide baseline data for species-specific GHG mitigation and challenge cattle-centric models in warming regions.

## INTRODUCTION

Climate change is an important environmental factor that negatively influences livestock, including dairy ruminants. With the high demand for milk, dairy farming tends to expand from temperate to tropical regions [1]. Prolonged high ambient temperature (HTa) has been reported in tropical areas, and this condition is associated with reduced milk production in dairy cows and goats [2]. This accelerates the perpetuating cycle of global warming by increasing greenhouse gas (GHG) emissions from dairy ruminants, which in turn increases the ambient temperature (Ta).

Carbon dioxide (CO₂) and methane (CH₄) are the two most potent GHGs generated from ruminant fermentation. Ruminal CH₄ has been reported to contribute approximately 6% of total anthropogenic GHG emissions [3]. In addition, the global warming potential over 100 years (GWP100) for CH₄ is approximately 28 times that of CO₂-equivalent [4]. In dairy cows, HTa has been demonstrated to increase enteric CH₄ emission yield by influencing the rumen fermentation patterns [5–7]. From retrospective data, it has been proposed that dry matter intake (DMI) is associated with CH₄ emissions under HTa conditions [8]. This relationship was supported by the effect that the feeding schedule had on the diurnal CH₄ emission pattern in dairy cows [5]. To our knowledge, the HTa conditions in most of those experiments were much lower than those in tropical conditions [2, 5, 7]. Further, it has been known that dairy goats, as small ruminants, also play a prominent role in responding to GHG emissions. The anthropogenic GHG emissions from livestock have been reported at approximately 7-18%. Of this, goats contributed approximately 4-5% of overall GHG emissions from livestock [9].

While numerous studies have examined HTa effects on CH₄ in dairy cows under controlled chambers or temperate conditions [5–8], data on natural seasonal exposure in goats, which are more heat-tolerant and increasingly important in tropical smallholder systems, remain scarce. Because GHG emissions from goats are lower than those from other livestock per unit of body weight (BW) [9] and because comparable information on the HTa effect on GHG emissions has not been demonstrated in dairy goats under natural ambient conditions, critical knowledge gaps persist regarding long-term acclimatization versus short-term diurnal effects in tropical environments. Most existing research focuses on cattle-centric models and acute heat stress protocols that do not fully represent the chronic, year-round high temperature–humidity index conditions typical of tropical regions or the superior adaptability of crossbred goats.

Given the current interest in reducing global warming and the effects of natural HTa on GHG emissions from dairy ruminants, the present experiment focused on the seasonal effect that represents long-term natural HTa exposure. The long-term natural conditions are considered the major external factor that determines acclimatization and adaptation. In addition, this study is novel in (1) using a true seasonal design representing long-term acclimatization, (2) employing respiratory chambers under both ambient and cooled conditions in the same animals, and (3) focusing on crossbred tropical goats rather than pure exotic breeds. The data provide a foundational baseline to help explain the superior physiological adaptability and production efficiency of goats relative to other livestock [2]. We hypothesized that the daily CO₂ or CH₄ emission patterns differ between winter and summer, but goats’ superior adaptability may prevent this response.

## MATERIALS AND ‘METHODS

### Ethical approval

All experimental procedures were reviewed, approved, and monitored by the Animal Ethics Committee of Can Tho University, Vietnam, under Animal Use Protocol No. CTU-AEC25003. The protocol adhered to the highest standards of animal welfare, including the principles of the 3Rs (Replacement, Reduction, and Refinement). Measures were implemented to minimize pain, distress, and discomfort through appropriate housing in individual metabolic cages that allowed normal postural adjustments and behaviors, provision of ad libitum access to feed and water, regular health monitoring by qualified veterinarians, and humane endpoints. Goats were purchased only from a reputable local farm with verified vaccination records and underwent thorough clinical examinations prior to enrolment to ensure good health status. All personnel involved in animal handling received training in laboratory animal science and ethical practices. The study complied with national and international guidelines for the care and use of farm animals in research, including those outlined by the Canadian Council on Animal Care and the American Veterinary Medical Association, ensuring scientific validity while prioritizing animal well-being. The ethics committee maintained regular oversight throughout the experimental periods, with provisions for immediate intervention if any adverse events occurred. This ethical framework ensured that the research balanced scientific objectives with responsible animal use.

### Study period and location

The study was conducted at Hoa An’s practical farm in Phung Hiep, Hau Giang, the third campus of Can Tho University, Can Tho, Vietnam.

### Study design

The study followed a completely randomized design with two treatment periods and six replicates per treatment. For each treatment period, six nulliparous goats were randomly allocated from vendors. The number of goats from each period was estimated using sample size calculations based on our previous results [2]. Two treatment periods included winter (December–February) and summer (March–May), which align with the cool and hot seasonal trends according to the Meteorological Department of Vietnam. Each experimental period lasted six weeks and included one week of adaptation (day 1-7). Specifically, the 24 h GHG emission patterns were measured with 2 chamber conditions in the middle of weeks 2 and 6. Ruminal fluid and blood collections were performed during week 3. The protocols for sample acquisition are outlined below.

### Animals, husbandry, and ethics approval

Twelve nulliparous crossbred mature female Saanen goats (Saanen × Bach Thao; 50%), aged 8–10 months with an average BW of 18.47 ± 1.18 kg and a body condition score of 2.5/5, were included in this experiment. The nulliparous goats were selected because they exhibited minimal exposure to circulating reproductive hormones, thereby precluding potential endocrinological confounding variables. The goats were purchased from a local farm with a good vaccination record and normal clinical examination results before enrollment to confirm their good health status. The goats were housed in individual metabolic cages at Ta and were provided with total mixed ration (TMR) and ad libitum access to freshwater. The experimental diet consisted of 80% corn silage and 20% concentrate (Table 1).

**Table 1:** Feed ingredients and chemical composition of the experimental diet (DM basis) based on the feed analysis from the total mixed ration experiment.

**Diet composition**	**Control**
Ingredients, %	
Corn silage	80
Broken rice	2.0
Soybean extraction meal	8.6
Rice bran	2.0
Coconut meal	7.1
Premix Vitamin-Mineral	0.1
Salt	0.2
Chemical compositions, %	
DM	88.4
OM	91.5
CP	12.3
EE	3.67
NDF	49.1
ADF	37.9
Ash	8.46
Na	8.37
K	12.0
Cl	0.410
S	0.336
DCAD, mEq/100g DM	19.6

DM = Dry matter; OM = Organic matter; CP = Crude protein; EE = Ether extract; NDF = Neutral detergent fiber; ADF = Acid detergent fiber; Na = Sodium; K = Potassium; Cl = Chloride; S = Sulfur; DCAD = Dietary cation–anion difference, calculated as [(Na + K) − (Cl + S)] and expressed as mEq/100 g DM. Vitamin-mineral premix supplied essential vitamins and trace minerals according to the manufacturer's recommendations.

### Physiological and environmental measurements

Ambient temperature and humidity were recorded on the experimental farm daily using a digital monitoring traceable barometer (6530-B, Traceable®-Cole-Parmer, Vernon Hills, IL, USA). The feeding schedule was twice daily (07:00 and 14:00), with TMR offered ad libitum (refusals not exceeding 10% of the offered feed). Daily feed and water intakes (WI) were measured by weighing the offered and refused TMR and drinking water. Respiratory rate (RR) and rectal temperature (Tr) were determined twice per week, morning and afternoon, including on the day of sampling collection. Measurement of Tr was performed by inserting a digital thermometer (digital clinical thermometer C202, Terumo, Tokyo, Japan) into the rectum, whereas RR was determined by counting chest excursions over a 1-minute interval. Both physiological parameters were recorded at 06:00 and 14:00 hours.

### Respiratory chamber measurements

In the middle of the second week (day 11), each goat was placed in a respiratory chamber for 24 h for acclimation. The CO_2_ and CH_4_ emissions were measured for 24 h, with every-minute gas measurements, from each goat under two conditions. While the current experiment compared different seasons as a proxy for the long-term HTa effect, the paired gas measurement methodology was explicitly designed to mitigate the short-term diurnal HTa effect. The first measurement was taken in the middle of the third week (day 18) within the respiratory chamber, which was maintained at ambient conditions. The second measurement was performed in the middle of the sixth week (day 39) using a 25°C air-conditioned respiratory chamber. Each goat was transferred from the individual metabolic cage to an individual respiration chamber 1 day before gas measurements, with the chamber door open for preparation, resulting in a confinement duration of 48 h per animal. The temperature and humidity from the respiratory chamber were measured using a Kestrel agriculture meter (Kestrel 3000 Weather Meter– Part #0830, Nielsen-Kellerman Company, Boothwyn, PA, USA). Measurements of ambient conditions inside the chamber were taken every 2 h during the daytime (06:00–18:00). The Ta and relative humidity (RH) values from each point were used for calculating the temperature–humidity index (THI), which was calculated according to NRC [10] using the following equation:

THI = (1.8 × Tdb + 32) − [(0.55 − 0.0055 × RH) × (1.8 × Tdb − 26.8)],

where Tdb represents the dry-bulb temperature (°C), and RH represents relative humidity (%).

### Feed sampling and analysis

In the present experiment, feed and refusal samples were collected and weighed daily, then divided into two portions. One portion was immediately dried in a forced-air oven at 105°C until a constant weight was achieved to determine the dry matter (DM) and DMI. The remaining portion was stored at −20°C until further chemical analysis. At the end of the experiment, all feed samples were thoroughly mixed, and a representative subsample was dried at 65°C overnight (approximately 12 h) and analyzed for DM, nitrogen, and ash according to AOAC [11]. Neutral detergent fiber and acid detergent fiber were determined following the previously described procedure by Van Soest [12].

### Respiratory chamber, gas sampling, and analysis

The respiratory chamber was designed as a closed cage equipped with an air conditioner to regulate the internal temperature, a feeding trough, and a water bucket. The chamber was made of 10-mm transparent acrylic panels (1.00 m long, 1.79 m high, and 1.87 m wide). Transparent materials were used to facilitate visual observation of animal behavior. All panels were sealed with silicone, except the front and back panels, which were attached to two flexible panels for opening. The chamber contained air inlets that would be installed at each corner [13]. The metabolic cage, designed to house the goats within the respiratory chamber, was constructed from stainless steel (0.8 m long, 1.28 m high, and 1.2 m wide). The floor was composed of 10-mm-thick wood panels, each 50 mm wide and spaced 15 mm apart, thereby providing a comfortable surface for the animal while allowing feces and urine to drop down. Feces would drop into the bottom tray, and a 15-mm hole at the bottom, beside the chamber, allowed urine to drain into a bucket. A ring blower (GR40-610, Dongnam Engineering Co., Ansan, Korea) was installed to draw air from the chamber through PVC pipes at a flow rate of approximately 350 L/min [13]. Based on our preliminary study, the specified airflow rate maintained goats within comfortable conditions and allowed for expression of normal behaviors, including resting, eating, drinking, and breathing. Outlet air from each chamber was filtered through an air filter, and the flow rate was measured using a thermal mass flow meter corrected for standard temperature and pressure. Airflow rate data measured by the thermal mass flow meter was transferred to a data logger and recorded on a computer at 4-min intervals. A sampling pump (Resun GF120, Taipei, Taiwan) was used to maintain continuous airflow through the system. A mixture of gas (Cylinder No. 46837) containing 0.19% CH₄, 1.9% CO₂, and 97.91% N₂ was used for calibration, with the recovery rate ranging from 97-103% for all chambers before each measurement. The background of CO₂ and CH₄ without animal that were measured 24 h were closed to zero gram per day, 0.0016 and 0.000 L/min. The gas samples were then passed through a dehumidifier containing CaSO₄ to remove the moisture. The concentrations of CO₂ and CH₄ were analyzed using a non-dispersive infrared gas analyzer (Model IR200, Yokogawa, Japan) with a measuring range of 0–2,000 ppm and a repeatability of 0.5% through a 6-mm-diameter pipe. The concentrations of CO₂ and CH₄ in the air samples were determined and transferred at 1-min intervals to the JIRAS HC System 2.0.1 (Type VA-3000, Horiba Stec Co., Kyoto, Japan).

### Plasma cortisol concentration

A blood sample (5 mL) was collected from the jugular vein on the last day of week 3 (day 21) at two sampling times, 06:00 and 14:00. The purpose of this measurement is to demonstrate the diurnal HTa effect on stress response [2] and to compare between seasons. To harvest plasma, blood was centrifuged at 12,000 × *g* at 4°C for 10 min, then kept under −80°C for cortisol. Plasma cortisol concentrations were determined using a commercial enzyme-linked immunosorbent assay kit (CBS-E18048G, CUSABIO, Houston, TX, USA), with an intra-assay variability of 7.3% relative to the standard curve. In this competitive assay, plasma cortisol competed with pre-coated antigen, such that stronger color development indicated lower plasma cortisol concentration. For analysis, 50 µL of each standard and sample were mixed with an equal volume of antibody in the wells and incubated at 37°C for 40 min. The wells were then washed three times with washing buffer, after which 100 µL of horseradish peroxidase (HRP)-conjugated reagent was added to each well and incubated at 37°C for an additional 40 min. After a second washing step, 90 µL of tetramethylbenzidine substrate was added and incubated at 37°C for 20 min. Finally, a stop solution was added, and the optical density was measured at 450 nm an ELISA microplate reader (BK-EL10C, BIOBASE, Shandong, China).

### Volatile fatty acids (VFAs) analysis

Rumen fluid samples were collected from goats on day 20 of the experiment using a stomach tube connected to a syringe. Approximately 10 mL of ruminal fluid was collected at 10:00 (approximately 3 h post-feeding). The purpose of this procedure is to present meal-related ruminal fermentation on VFAs. To minimize oral secretions and ensure a representative ruminal sample, a coaxial double-tube sampling method was deployed. A rigid outer sheath (22 mm internal diameter) was anchored via an oral speculum to shield the tract. A flexible inner collection catheter (10 mm outer diameter, 110 cm length) was then advanced through the lumen directly into the ruminal cavity. The pH of the samples was measured immediately using a pH meter (pH221; Lutron, Taipei, Taiwan). The samples were then filtered through two layers of cheesecloth, and 1 mL of 6 N HCl was added to preserve them. Finally, the samples were stored at −20°C until further analysis for ammonium nitrogen (NH_3_-N) and VFAs. The concentration of NH₃-N was determined by distillation with boric acid absorption and titration with 0.1 N H₂SO₄, following the Kjeldahl procedure by AOAC [14], and ruminal VFAs were determined according to Thanh *et al*. [15]. For VFA analysis, 5 mL of rumen fluid was thawed and mixed with 1 mL of 25% metaphosphoric acid and formic acid (3:1, v/v) in a centrifuge tube, and allowed to precipitate for 30 min. The mixture was then centrifuged at 10,000 × *g* for 15 min, and the clear supernatant was collected for gas chromatography analysis. VFAs were determined using a gas chromatograph (Thermo Scientific Trace 1310 GC system, Waltham, MA, USA) equipped with a flame ionization detector. Injector and detector temperatures were maintained at 220°C. Aliquots of 1 µL were injected with a split ratio of 10:1 into a 30 m × 0.25 mm × 0.25 µm Nukol fused-silica capillary column (#24107, Supelco, Sigma–Aldrich, St. Louis, MO, USA). Helium was used as the carrier gas at a flow rate of 1 mL/min, with an initial oven temperature of 80°C. The oven temperature was held at 80°C for 1 min, increased to 180°C at 20°C/min and held for 1 min, and then increased at a rate of 10°C/min to a final temperature of 200°C, with a total run time of 14 min. The VFA peaks were identified by comparing their retention times with those of external standards, including acetic, propionic, butyric, iso-butyric, and iso-valeric acids (Sigma–Aldrich, Louis, MO).

### Statistical analysis

All data are presented as the mean ± standard error of the mean. Two data sets were assessed for the normality and homogeneity of variance using the Shapiro–Wilk test and Levene’s test, respectively. The difference of two data sets that met these assumptions were analyzed using a Student’s t-test for one-factor analysis. A linear mixed-effects model was used for repeated-measures data across experimental period, with season and chamber (or ambient and 25 °C conditioning within respiratory chamber) as fixed effects as follows.

Yijk = μ + Si + Cj + (S × C)ij + Gk + εijk,

where Yijk represents the observed response variable, μ represents the overall mean, Si represents the fixed effect of season, Cj represents the fixed effect of chamber, (S × C)ij represents the treatment × chamber interaction, Gk represents the random effect of goat, and εijk represents the residual error. The Bonferroni test was performed as a post hoc analysis to assess differences between groups. Differences were considered significant at p < 0.05.

## RESULTS

### Seasonal conditions and the HTa effect on behavioral and physiological parameters

**Figure 1:**
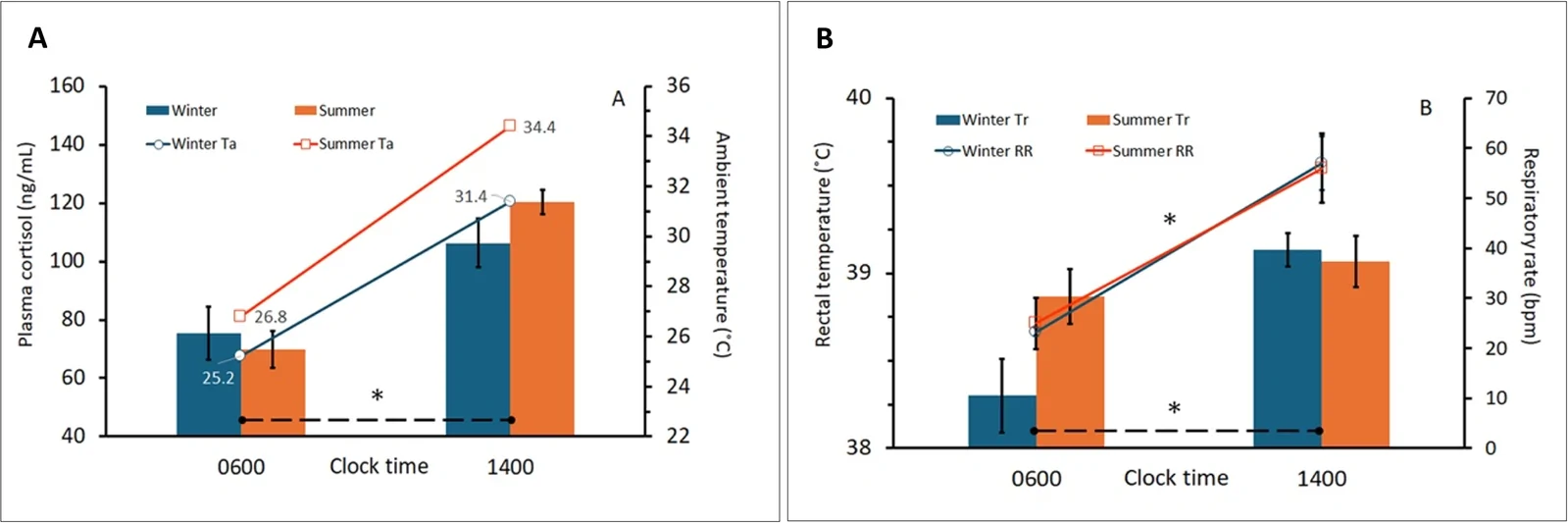
Effect of long-term HTa exposure (winter and summer) on physiological responses. (A) Morning (0600) and afternoon (1400) ambient temperature (line, right x-axis) and plasma cortisol (bar, left x-axis). (B) Morning (0600) and afternoon (1400) respiratory rate (line, right x-axis) and rectal temperature (bar, left x-axis). * Significant effect of season (p < 0.05).

During the experimental period, ambient conditions were recorded from morning to afternoon in winter and summer months. The average morning and afternoon Ta, RH, and THI from both seasons were presented in Table 2. The average summer and winter Ta difference (∆Ta) from morning and afternoon was 1.6 and 3.0°C, respectively (Figure 1A). During the experimental periods, plasma cortisol, RR, and Tr from the afternoon were significantly higher than those from the morning (Figures 1A and B; F1,10 = 59.3, 64.8, and 32.9, respectively; p < 0.05). However, there was no seasonal effect on plasma cortisol, Tr, and RR (F1,10 = 0.2, 1.5, and 0.003, respectively; p > 0.05).

**Table 2:** The average ambient conditions from the environmental farm during winter and summer.

**Season**	**Winter**		**Summer**	
	**Morning**	**Afternoon**	**Morning**	**Afternoon**
Amb Ta (°C)	23.58 ± 0.22	30.10 ± 0.20	26.89 ± 0.14	31.97 ± 0.24
Amb RH (%)	88.0 ± 0.7	67.5 ± 0.6	89.2 ± 0.3	71.2 ± 1.0
Amb THI	73.4 ± 0.4	81.3 ± 0.3	79.1 ± 0.2	84.7 ± 0.2

Amb = Ambient; Ta = Temperature; RH = Relative humidity; THI = Temperature–humidity index. Data are presented as mean ± standard error (SE). THI was calculated using ambient temperature and relative humidity.

Across the winter and summer periods, the average DMI and WI in the current experiment did not differ significantly (Table 3; T10=0.98 and T10=0.02, respectively; p > 0.05). There was no effect of season on these behaviors when DMI and WI were both normalized by BW (Table 3; T10=0.42 and T10=0.98, respectively; p > 0.05), and there was no effect of season on the DMI and WI ratios (Table 2; T10=1.25; p > 0.05). Although eating and drinking were not influenced by season, there was evidence of a seasonal effect on rumen fermentation. Ruminal VFA and NH_3_-N were influenced by the different seasons (Table 3; T10=3.86 and 3.17, respectively; p < 0.05). Moreover, the ruminal concentration ratio of acetate to propionate from summer was higher than that from winter (Table 3; T10=2.42; p < 0.05).

### Seasonal conditions and the HTa effect on respiratory chamber conditions

**Figure 2:**
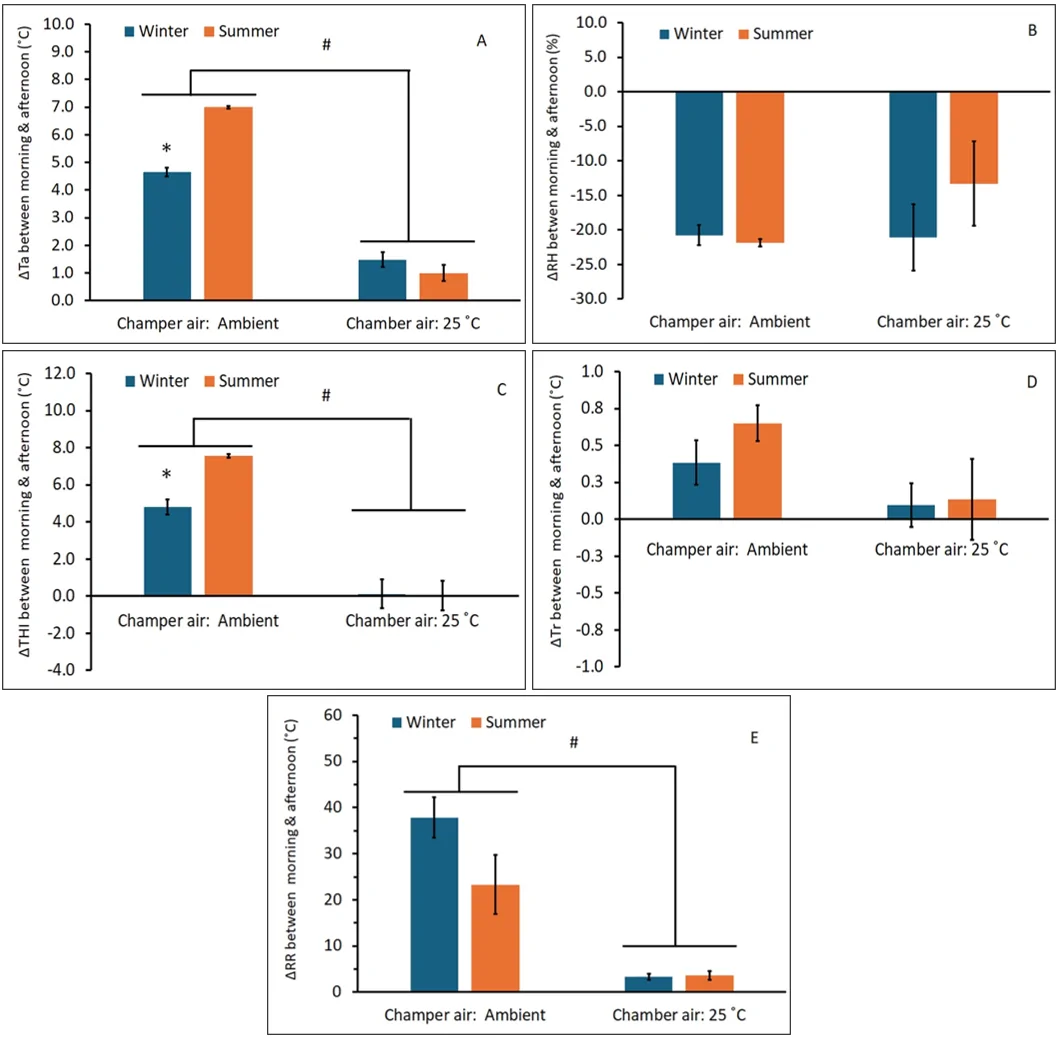
The difference between morning and afternoon ambient temperature (Ta), relative humidity (RH), temperature–humidity index (THI), rectal temperature (Tr), and respiratory rate (RR) from two chamber conditions of GHG measurement. (A) ∆Ta (B) ∆RH, (C) ∆THI (D) ∆Tr and (E) ∆RR. * Significant effect of season (p < 0.05). # Significant effect of chamber (p < 0.05).

GHG measurements were performed under ambient (Amb) and 25°C air-conditioned conditions. Table 3 demonstrates Ta, RR, and THI within the respiratory chamber under the first and the second (25°C) conditions. Under the first condition, there were significant effects of daytime and season on Ta, RH, and THI (Table 4; F1,10=5112 & 47, 790 & 23 and 784 & 27, respectively; p < 0.01). Goats from the first condition exhibited that the Tr and RR were both influenced by daytime (Table 4; F1,10=29 and 62, respectively; p < 0.01). However, only Tr from the first condition was influenced by season (Table 4; F1, 10 = 5; p = 0.049). These ambient conditions and physiological responses differed from those in the second condition, in which the temperature chamber was maintained at 25°C. Under the second condition, there were significant effects of daytime on Ta and RH (Table 4; F1, 10 = 40 and 20, respectively; p < 0.01), but not on THI (Table 4; F1, 10 = 0.03; p = 0.86). Because of the air conditioning, there were no seasonal effects on Ta, RH, and THI (Table 4; F1, 10 = 0.02, 2.13, and 0.20, respectively; p > 0.05). In addition, there was still a daytime effect on RR (Table 3; F1, 10 = 38; p < 0.05) and a season effect on Tr (Table 4; F1, 10 = 14; p < 0.05) under an air-conditioned setting. To analyze the seasonal and chamber effects of daytime on GHG measurement, the differences of Ta, RH, and THI values between morning and afternoon (∆Ta, ∆RH, and ∆THI, respectively) in the chamber were calculated (Figures 2A–C). There were significant effects of the chamber on ∆Ta and ∆THI (Figures 2A and 2C; F1, 10 = 607 and 125, respectively; p < 0.01), but not ∆RH (Figure 2B; F1, 10 = 1.1; p = 0.4). Under ambient conditions, there were higher daily ∆Ta and ∆THI during summer than during winter (Figures 2A and 2C; T10 = 7.8 and 3.3, respectively; p < 0.01). When similar differences of Tr and RR (∆Tr and ∆RR) were calculated and analyzed (Figures 2D and 2E), only ∆RR values from the 25°C chamber were lower than those from the Amb chamber (Figures 2E; F1, 10 = 48; p < 0.01).

**Table 3:** Behavioral and physiological indexes across the winter and summer periods.

**Parameter**	**Winter**	**Summer**	**p-value**
DMI (g/day)	355 ± 39.17	402 ± 27.36	0.35
DMI per BW (g/kg/day)	18.3 ± 1.69	19.4 ± 2.22	0.68
WI (mL/day)	1685 ± 88	1683 ± 30	0.98
WI per BW (mL/kg/day)	88 ± 5.92	81 ± 4.68	0.35
DMI:WI per BW ratio	0.21 ± 0.02	0.24 ± 0.02	0.24
Ruminal fluid pH	6.76 ± 0.07	6.94 ± 0.06	0.09
Ruminal NH₃-N (mg/dL)	30.13 ± 1.02	26.13 ± 0.14	0.01
Total VFA (mM)	53.2 ± 0.72	57.8 ± 0.97	0.01
Acetate (% mole)	59 ± 1.75	65 ± 2.65	0.09
Propionate (% mole)	27 ± 2.78	20 ± 1.57	0.06
Butyrate (% mole)	13 ± 1.90	14 ± 2.08	0.78
Acetate:propionate ratio	2.3 ± 0.22	3.3 ± 0.37	0.04

DMI = Dry matter intake; BW = Body weight; WI = Water intake; DMI:WI = Dry matter intake-to-water intake; NH₃-N = Ammonia nitrogen; VFA = Volatile fatty acid. Data are presented as mean ± standard error (SE). Ruminal NH₃-N is expressed as mg/dL of ruminal fluid, total VFA as mM, and individual VFA as molar percentage (% mole). Acetate:propionate ratio was calculated from the molar proportions of acetate and propionate. p < 0.05 was considered statistically significant.

**Table 4:** Ambient conditions, rectal temperature and respiratory rate within gas measurement chamber.

**Season**	**Winter**		**Summer**		**SEM**	**Effects**		
	**Morning**	**Afternoon**	**Morning**	**Afternoon**		**S**	**T**	**S × T**
Amb Ta (°C)	25.15 ± 0.25	29.80 ± 0.40	26.60 ± 0.18	33.60 ± 0.22	0.06	0.01	0.01	0.01
Amb RH (%)	91.0 ± 0.0	70.3 ± 1.5	88.1 ± 0.4	66.2 ± 0.1	0.54	0.01	0.01	0.43
Amb THI	76.3 ± 0.4	81.1 ± 0.8	78.4 ± 0.3	86.0 ± 0.3	0.16	0.01	0.01	0.01
Amb Tr (°C)	38.8 ± 0.1	39.1 ± 0.1	38.9 ± 0.1	39.5 ± 0.1*	0.21	0.049	0.01	0.20
Amb RR (bpm)	23 ± 1	61 ± 5	29 ± 6	52 ± 6	2.74	0.80	0.01	0.09
25°C Ta (°C)	23.97 ± 0.41	25.45 ± 0.24	24.27 ± 0.42	25.27 ± 0.34	0.14	0.90	0.01	0.25
25°C RH (%)	87.42 ± 1.9	66.37 ± 5.5	78.23 ± 3.8	64.93 ± 2.8	2.75	0.18	0.01	0.34
25°C THI	73.97 ± 0.8	74.10 ± 0.7	73.59 ± 1.0	73.64 ± 0.4	0.39	0.66	0.86	0.93
25°C Tr (°C)	38.5 ± 0.1	38.6 ± 0.1	38.9 ± 0.2	39.0 ± 0.2	0.11	0.01	0.50	0.87
25°C RR (bpm)	19 ± 1	23 ± 1	19 ± 1	22 ± 1	0.40	0.65	0.01	0.77

S = Season; T = Time of day; S × T = Season × time of day interaction; SEM = Standard error of the mean; Amb = Ambient; Ta = Temperature; RH = Relative humidity; THI = Temperature–humidity index; Tr = Rectal temperature; RR = Respiratory rate; bpm = Breaths per minute. Values are presented as mean ± standard error (SE). Measurements at 25°C were obtained under thermoneutral environmental conditions. *Different from the corresponding winter afternoon value (p < 0.05). Statistical significance was declared at p < 0.05.

### Seasonal conditions and the HTa effect on DMI, WI, and GHG emissions

On the day of gas measurement, there were no seasonal and chamber effects on DMI (Table 5; F1, 10 = 0.92 and 0.12, respectively; p > 0.05). When DMI was normalized to BW, there was a significant seasonal effect but no chamber effect (Table 5; F1, 10 = 6.98; p < 0.05 and F1,10 = 0.13; p > 0.05). Moreover, the chamber effect was demonstrated by the WI and WI/BW (Table 5; F1, 10 = 124 and 15, respectively; p < 0.05), with the WI and WI/BW from the 25°C condition lower than those from the Amb condition. Moreover, there were no seasonal and chamber effects on the ratio of DMI:WI per BW (Table 5; F1, 10 = 0.67 and 4.24, respectively; p > 0.05).

**Figure 3:**
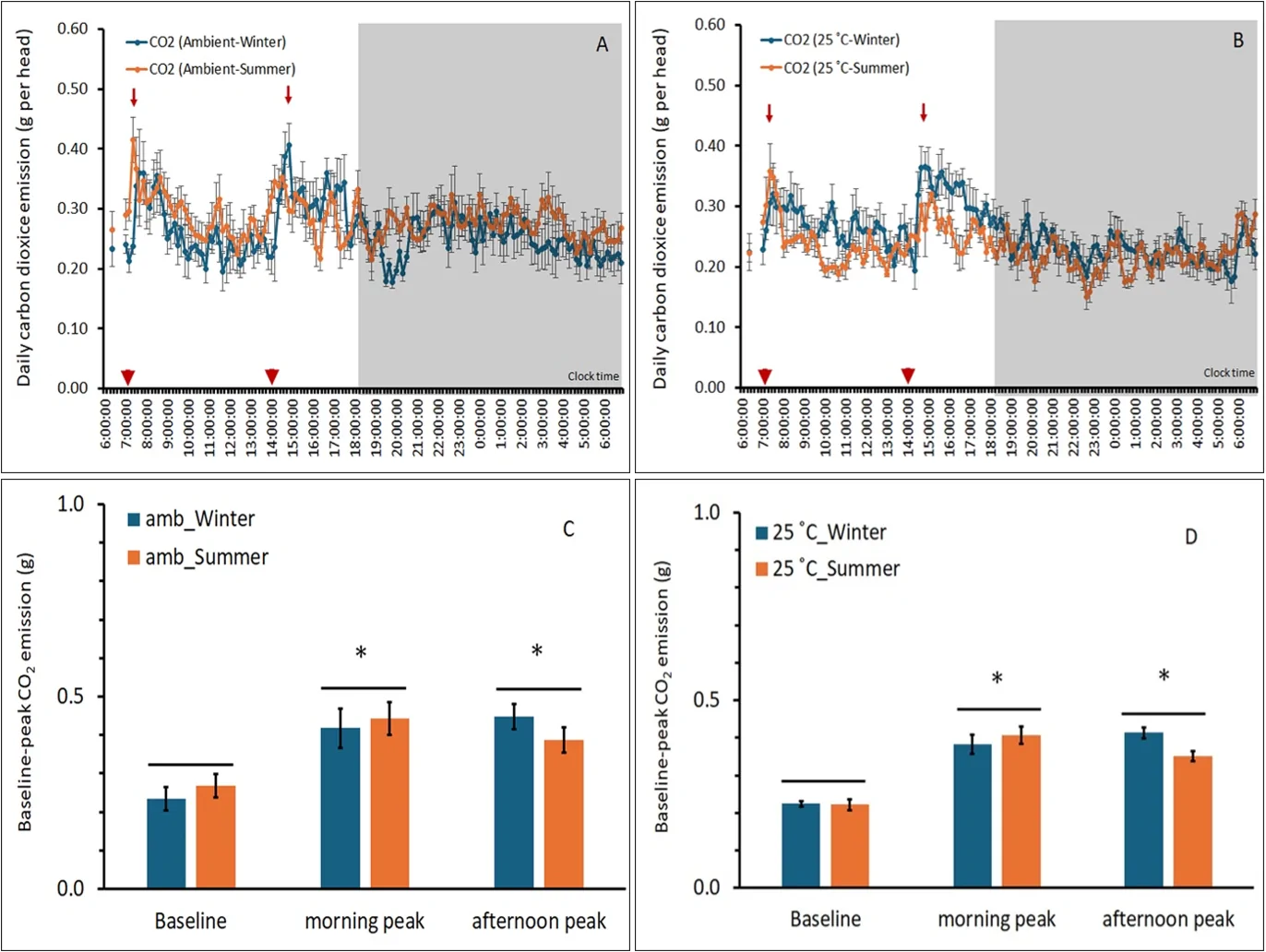
Daily carbon dioxide emission pattern between winter and summer periods from (A) ambient conditions and (B) 25°C conditions and peak analysis (C and D). The arrowheads from graphs A and B indicate feeding times, and the arrows represent the highest morning and afternoon peaks. * Significantly higher peaks from baseline (p < 0.05).

**Figure 4:**
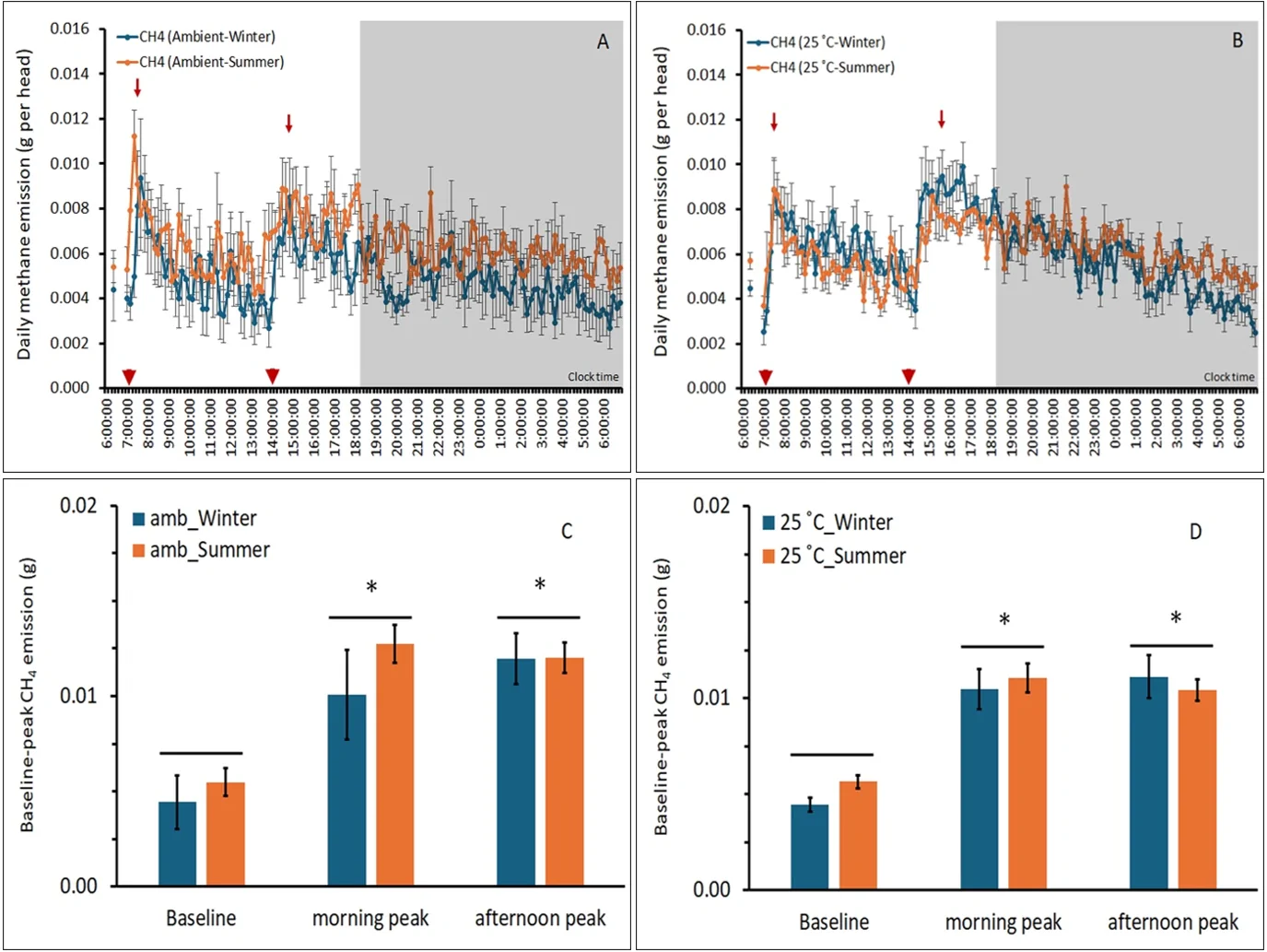
Daily methane emission pattern between winter and summer periods from (A) ambient conditions and (B) 25°C conditions and peak analysis (C and D). The arrowheads from graphs A and B indicate feeding times, and the arrows represent the highest morning and afternoon peaks. * Significantly higher peaks from baseline (p < 0.05).

The emission patterns of CO₂ and CH₄ were analyzed and are presented in Table 4, including daily emissions, emission yields, and daytime and nighttime emissions from the current experiment. Daily CO₂ and CH₄ emissions (Table 5; F1, 10 = 0.01 and 3.2, respectively; p > 0.05) and emission yields (Table 5; F1, 10 =1.5 and 0.2, respectively; p > 0.05) were not influenced by season and measurement conditions. Only nighttime CO₂ emissions were affected by measurement conditions (Table 5; F1, 10 = 8.3, p < 0.05). Moreover, pairwise comparisons revealed that nighttime CO₂ emissions in the 25 °C condition were significantly lower than those in the ambient condition (T10 = 2.97; p < 0.05). When analysis was performed in relation to feeding, there were no seasonal and measurement condition effects on CO₂ (Table 5; F1, 10 = 0.2 and 3.5, respectively; p > 0.05) or CH₄ (F1, 10 = 3.5 and 4.2, respectively; p > 0.05). In the current experiment, the 24 h emissions pattern for both CO₂ (Figures 3A and B) and CH₄ (Figures 4A and B) was graphed by averaging every 10 mins. When analysis was performed using the highest peak from baseline, morning, and afternoon (Figures 3A and B and Figures 4A and B, arrows), there were significantly higher values from the morning and afternoon peaks than from the basal peak for CO₂ emission from the Amb (Figure 3C; F2, 10 = 49.6, p < 0.05) and 25°C conditions (Figure 3D; F2, 10 = 83.3, p < 0.05). Similarly, CH₄ emissions were significantly higher (Figures 4C and D; F1, 10 = 52.5 and 77.7, p < 0.05).

**Table 5:** Dry matter and water intake from two chamber conditions of GHG measurement. GHG emission patterns for (middle part) carbon dioxide and (lower part) methane.

**Chamber**	**Ambient**		**25°C Condition**		**SEM**	**Effects**		
	**Winter**	**Summer**	**Winter**	**Summer**		**S**	**C**	**S × C**
DMI (g/day)	450 ± 43	390 ± 44	411 ± 13	404 ± 38	26.67	0.36	0.74	0.50
DMI per BW (g/kg/day)	23 ± 1.8	18 ± 1.2	21 ± 1.5	19 ± 1.2	1.11	0.02	0.72	0.44
WI (mL/day)	1570 ± 82	1627 ± 68	1222 ± 86	953 ± 99	32.49	0.36	0.01	0.01
WI per BW (mL/kg/day)	70.5 ± 6.9	91.0 ± 6.2	60.9 ± 8.8	51.9 ± 6.6	4.52	0.48	0.01	0.05
DMI:WI per BW ratio	0.35 ± 0.12	0.21 ± 0.03	0.40 ± 0.08	0.43 ± 0.11	0.05	0.43	0.07	0.14
Daily CO₂ (g/day)	377.7 ± 40	407.0 ± 32	366.9 ± 13	333.1 ± 13	16.7	0.94	0.10	0.21
CO₂ Yield (g/kg DMI)	875 ± 138	1077 ± 89	895 ± 37	863 ± 96	13.27	0.35	0.37	0.21
Daytime CO₂ (g/12 h)	199.1 ± 21	208.6 ± 18	203.7 ± 7	176.8 ± 5	10.1	0.56	0.36	0.23
Nighttime CO₂ (g/12 h)	178.6 ± 20	198.3 ± 14	163.2 ± 7	156.3 ± 9^#^	7.1	0.70	0.02	0.21
Morning feeding CO₂ (g)	53.11 ± 7	62.28 ± 3	53.67 ± 4	47.87 ± 1	2.86	0.69	0.12	0.09
Afternoon feeding CO₂ (g)	73.32 ± 6	61.95 ± 5	65.30 ± 6	50.49 ± 6	3.20	0.09	0.07	0.63
Daily CH₄ (g/day)	7.20 ± 1.7	9.16 ± 1.0	8.74 ± 0.9	8.87 ± 0.5	0.95	0.23	0.65	0.51
CH₄ Yield (g/kg DMI)	16.60 ± 4.5	23.92 ± 2.2	21.18 ± 1.8	23.59 ± 3.8	7.92	0.28	0.32	0.85
Daytime CH₄ (g/12 h)	3.94 ± 0.9	4.86 ± 0.6	4.96 ± 0.6	4.48 ± 0.3	0.56	0.60	0.69	0.39
Nighttime CH₄ (g/12 h)	3.26 ± 0.8	4.30 ± 0.5	3.78 ± 0.3	4.39 ± 0.2	0.40	0.09	0.58	0.71
Morning feeding CH₄ (g)	1.15 ± 0.3	1.46 ± 0.2	1.25 ± 0.2	1.08 ± 0.1	0.17	0.57	0.57	0.34
Afternoon feeding CH₄ (g)	1.23 ± 0.2	1.40 ± 0.1	2.14 ± 0.3	1.61 ± 0.2	0.21	0.29	0.09	0.27

S = Season; C = Chamber condition; S × C = Season × chamber condition interaction; SEM = Standard error of the mean; DMI = Dry matter intake; BW = Body weight; WI = Water intake; DMI:WI = Dry matter intake-to-water intake ratio; CO₂ = Carbon dioxide; CH₄ = Methane. Values are presented as mean ± standard error (SE). CO₂ and CH₄ emissions are expressed as g/day unless otherwise indicated. CO₂ and CH₄ yield are expressed as g/kg DMI. Daytime and nighttime emissions represent cumulative gas production over 12 h. Morning and afternoon feeding emissions represent cumulative gas production during the respective post-feeding measurement periods. #Different from the corresponding ambient condition within the same season (p < 0.05). Statistical significance was declared at p < 0.05.

## DISCUSSION

### HTa effects on GHG emissions and rumen fermentation decoupling

This study revealed the effect of HTa on GHG emissions in crossbred dairy goats under tropical conditions. The experiment focused on long-term HTa exposure between winter and summer months as a seasonal effect and on short-term HTa exposure between morning and afternoon. In addition, gas measurements were performed in a respiratory chamber under Amb and air-conditioned conditions (25°C). The latter condition aimed to attenuate the short-term HTa effect. While the seasonal effect could not influence GHG emissions, the short-term HTa attenuation decreased the nighttime CO₂ emissions. The effect was, in part, described by the HTa effect on water-drinking behavior. This study provides the first evidence from tropical goats under natural seasonal conditions that long-term HTa can decouple rumen fermentation shifts from enteric GHG emissions.

### Physiological responses to seasonal and diurnal HTa

The meteorological data from the current study revealed typical tropical conditions, indicating prolonged HTa and RH. With short-term Ta transition from morning to afternoon (Figure 1A), there was a significant increase in plasma cortisol levels under the current HTa conditions, suggesting that goats were in the second degree of heat stress [2]. This is supported by the significant increase in Tr and RR. The current experimental conditions forced the THI values to exceed 80 during the daytime across seasons, with an afternoon ∆Ta of 3.0°C (Figure 1A). However, this ∆Ta degree was lower than our previous report (∆Ta = 5.5°C; [16]). Although the current experiment did not demonstrate a seasonal effect of HTa on the plasma cortisol levels, such an effect was observed in the morning Tr (Figure 1B). In addition, DMI and WI were not significantly lower under the current heat stress stage. This was different from our previous findings, which showed that the seasonal effect on eating was ∆Ta = 5.5°C [16]. The physiological responses of HTa from the current experiment were consistent with our previous information. They emphasized that long-term or seasonal HTa effects did not influence eating and drinking behavior at a seasonal ∆Ta of 3.0°C, but rather at 5.5°C, as previously reported [2, 16].

### Impact of seasonal HTa on ruminal fermentation patterns

We demonstrated in the current study that seasonal HTa influenced the ruminal fermentation patterns. At a comparable DMI, the WI ratio, total ruminal VFA concentration, and acetate to propionate ratio increased, whereas the NH₃-N concentrations decreased. Heat stress has been shown to negatively affect rumen function, including blood supply and fermentation patterns. In dairy cows and goats, rumination time was negatively correlated with THI [17, 18]. During heat stress conditions, saliva secretion and ruminal passage rate were decreased. These responses could modify the ruminal pH and increase digesta retention time [19, 20]. Moreover, the rumen microbiome has been shown to be modified under acute and chronic heat stress in dairy goats [21, 22]. Because seasonal HTa did not change eating and drinking behaviors, the seasonal HTa effect that modified the rumen fermentation pattern in the present experiment can be explained by its effects on rumination behavior, rumen motility, digesta passage rate, and the ruminal microbiome.

### Chamber conditions and short-term HTa attenuation effects

Because the ambient conditions in tropical areas typically include high temperatures, the year-round THI was above 80 in every month [2, 16]. Measurements of daily GHG emissions using the respiratory chamber unit were performed under two conditions: Amb and 25°C. Using respiratory chambers under both ambient and cooled conditions within seasons represents a powerful approach to disentangle chronic seasonal acclimatization from acute heat load effects from diurnal HTa. We hypothesized that GHG emission patterns could be changed when short-term HTa conditions or daytime HTa (Figure 1A) were attenuated on the measurement day (Figure 2). As expected, both Ta and THI decreased significantly with conditioning to 25°C (Figures 2A and 2C). The chamber effect was also demonstrated through drinking behavior, where WI and WI/BW from the 25°C condition were lower than those from the Amb condition, and only nighttime CO₂ emission was attenuated in summer (Table 4). The chamber effect on nighttime CO₂ emissions might be explained by the lower WI, which could influence salivary secretion, ruminal retention time, and fermented gas production [19], suggesting that attenuation of daytime HTa could decrease ruminal CO₂ production. Further experiments are needed to confirm the short-term HTa attenuation on the relationship between WI and nighttime CO₂ emission.

### Implications for CH₄ emissions and limitations

With the current seasonal ∆Ta at 3.0°C, there were no seasonal HTa and chamber effects on the CO₂ and CH₄ daily emissions. This is inconsistent with earlier reports that HTa-induced heat stress increased CH₄ emissions in dairy cows [5–6]. Increased CH₄ emission yield may be influenced in part by the HTa effect that decreased the DMI relative to CH₄ emission [7]. The seasonal HTa effect, which increases CH₄ emission, has been recorded in sheep but not in goats [23]. Contradictory information was also reported from non-lactating crossbred dairy cows in a climatic chamber exposed to HTa, where CH₄ emissions decreased [24]. In principle, daily CH₄ emissions and emission patterns are regulated by a multifactorial control mechanism. While gastrointestinal tract motility and microbiome are direct effects of HTa on emission [17–22], dietary types (roughage quality) and consumption behaviors (eating and drinking) are indirect effects of HTa, and feed rations (concentrate and roughage ratio or different formulation) are the external factors that could influence fermentation pattern and subsequently GHG production [18, 25–29]. Although we could see the effect of season on total VFA and acetate: propionate ratio, our results, which failed to demonstrate a seasonal HTa effect on CH₄ emission, emphasize the variability in outcomes across different HTa conditions and suggest that goats may already be near their adaptive threshold under existing climate scenarios. It does not appear that our GHG measurement technique was limited. However, the system clearly showed the effects of feeding-related CO₂ and CH₄ emissions (Figures 3 and 4), and we also demonstrated the chamber effect of CO₂ emissions. Furthermore, because goats have greater adaptability than other ruminants, i.e., cows and sheep [9, 30], the year-round HTa under current tropical conditions may be another environmental factor driving acclimatization in goats. Therefore, the seasonal HTa effect on CH₄ emission was not observed under the current seasonal ∆Ta at 3.0°C. Further experiments are required with higher summer Ta or high seasonal HTa (∆Ta > 5.5°C, [16]) and with different ruminant species with varying heat tolerance. The major limitation of the present investigation is the low degree of HTa difference; however, we also consider that, under the natural, uncontrolled conditions of Southeast Asia, the results can provide greater fidelity and contribute to the physiological responses of HTa in dairy ruminants [2].

## CONCLUSION

The current study demonstrated that seasonal HTa under tropical conditions altered ruminal fermentation patterns in non-lactating crossbred Saanen goats, evidenced by increased total VFA concentration, elevated acetate:propionate ratio, and decreased NH₃-N, without affecting DMI or WI. Despite these fermentation shifts and confirmed heat stress indicators (elevated afternoon plasma cortisol, Tr, and RR with THI >80), daily CO₂ and CH₄ emissions remained comparable between winter and summer seasons. However, short-term attenuation of daytime HTa to 25°C in respiratory chambers significantly reduced nighttime CO₂ emissions, linked to modified drinking behavior.

These findings highlight the superior heat adaptability of crossbred tropical goats, suggesting that current seasonal HTa levels (ΔTa ≈ 3.0°C) may not substantially elevate enteric GHG emissions in small ruminant systems. This supports targeted management strategies, such as optimized drinking water access and selective breeding for microbiome resilience, to maintain production efficiency while contributing to climate-smart livestock practices in warming tropical regions.

Key strengths include a true seasonal design with long-term acclimatization, paired respiratory chamber measurements under ambient and cooled conditions in the same animals, and comprehensive integration of physiological, fermentation, and emission data under natural tropical conditions.

The major limitation is the relatively moderate seasonal ΔTa (3.0°C), which may not fully represent more extreme future climate scenarios (e.g., ΔTa >5.5°C). Additionally, the focus on non-lactating goats limits direct extrapolation to high-producing lactating animals.

Further experiments are warranted with higher summer Ta, lactating goats, and different ruminant species varying in heat tolerance. Detailed metagenomic and metabolomic profiling of the rumen microbiome will elucidate mechanisms underlying the observed decoupling of fermentation changes from GHG emissions and identify microbial biomarkers for heat resilience.

In conclusion, tropical crossbred dairy goats exhibit remarkable physiological and microbial adaptability that mitigates the impact of seasonal HTa on enteric GHG emissions. These species-specific insights challenge cattle-centric models and provide a foundational baseline for accurate GHG accounting, welfare improvement, and sustainable livestock production in the face of global warming.

## DATA AVAILABILITY

The data generated during the study are included in the manuscript.

## GENERATIVE AI DECLARATION

The authors declare that no generative artificial intelligence or AI-assisted technologies were used in the writing, analysis, or preparation of this manuscript.

## AUTHORS’ CONTRIBUTIONS

ST and NTCP: Conceptualization, methodology, and writing – original draft. NTCP and ST: Writing – review and editing. NTCP, TTT, and NT: Data curation and writing – original draft. ST, NT, and NC: Supervision, data curation, and writing – original draft. All authors have read and approved the final manuscript.
